# Summer activity patterns among teenage girls: harmonic shape invariant modeling to estimate circadian cycles

**DOI:** 10.1186/1740-3391-10-2

**Published:** 2012-05-06

**Authors:** Semhar Ogbagaber, Paul S Albert, Daniel Lewin, Ronald J Iannotti

**Affiliations:** 1 Biostatistics and Bioinformatics Branch, Division of Epidemiology Statistics and Prevention Research, Eunice Kennedy Shriver National Institute of Child Health and Human Development, 6100 Executive Blvd, Bethesda, MD, 20892, USA; 2 National Center on Sleep Disorders Research, National Heart Lung and Blood, 6701 Rockledge Drive, Bethesda, MD, 20892, USA; 3 Prevention Research Branch, Division of Epidemiology Statistics and Prevention Research, Eunice Kennedy Shriver National Institute of Child Health and Human Development, 6100 Executive Blvd, Bethesda, MD, 20892, USA

**Keywords:** Adolescent medicine, Circadian rhythms, Obesity, Phase-shift, Statistical methods

## Abstract

**Background:**

Physical activity as measured by activity counts over short time intervals across a 24 h period are often used to assess circadian variation. We are interested in characterizing circadian patterns in activity among adolescents and examining how these patterns vary by obesity status. New statistical approaches are needed to examine how factors affect different features of the circadian pattern and to make appropriate covariate adjustments when the outcomes are longitudinal count data.

**Methods:**

We develop a statistical model for longitudinal or repeated activity count data that is used to examine differences in the overall activity level, amplitude (defined as the difference between the lowest and highest activity level over a 24 hour period), and phase shift. Using seven days of continuous activity monitoring, we characterize the circadian patterns and compare them between obese and non-obese adolescent girls.

**Results:**

We find a statistically significant phase delay in adolescent girls who were obese compared with their non-obese counterparts. After the appropriate adjustment for measured potential confounders, we did not find differences in mean activity level between the two groups.

**Conclusion:**

New statistical methodology was developed to identify a phase delay in obese compared with non-obese adolescents. This new approach for analyzing longitudinal circadian rhythm count data provides a useful statistical technique to add to the repertoire for those analyzing circadian rhythm data.

## Introduction

The purpose of this study was to characterize circadian rhythms patterns of activity among adolescent girls ages 15 to 16. Numerous health benefits have been associated with adolescent lifestyles that include moderate to vigorous physical activity (PA) [1]. Furthermore, adolescent PA tracks into adulthood and relates to adult obesity [2-4]. However, most adolescent girls do not meet recommendations for daily PA [5] and levels of PA decrease in girls during adolescence [6-8]. Changes in sleep health and circadian regulation have also been associated with decreased physical activity as well as health problems (e.g., obesity and diabetes), and decrements in quality of life and neurocognitive function [9-14].

In general the development of methodology to characterize changes in physical activity and to estimate circadian rhythms and the timing of sleep has the potential to provide insight into the causes of disease and could serve as a tool to evaluate treatment outcomes. In this particular case, characterizing circadian patterns in adolescent girls may provide guidance in targeting particular PA lifestyles or times of day most conducive to intervention. Although there is evidence for objective differences in the daily quantity of vigorous physical activity engaged in by normal weight versus overweight children and adolescents [15,16], less is known about how PA in overweight and normal adolescents may differ on other dimensions, such as time of day for peak activity, differences in periods of peak and minimal activity, and how PA fits within their daily rhythms.

The NEXT Generation Health Study provides a unique resource for characterization of adolescent PA and also provides an opportunity to examine the role of obesity on the circadian rhythm of PA. Specifically, are circadian PA patterns different across optimal weight and overweight groups?

In order to address these questions, we developed a new statistical modeling approach that allows us to characterize the effect of obesity on important features of the circadian rhythm. The proposed methodology allowed for analyses with and without adjustments for demographic variables. We proposed a shape invariant model for the effects of covariates on the circadian rhythm patterns in longitudinal activity (count) measurements. This modeling approach assumes that all subjects have the same underlying circadian pattern with differences across individuals and subgroups being reflected by changes in the mean, amplitude and phase shift of the underlying circadian pattern in activity that can also be viewed as changes in sleep period timing.

The study of circadian rhythms is common in the biological and social sciences [17] and is becoming increasingly important for health as circadian clock genes [18] have been identified in both neural and non-neural tissue. Coordination of these clock genes through the body may be critical for metabolic function, immunity and tissue repair as well as neurocognitive function. While the health effects of circadian regulation have only been studied in recent years, there is a longer history of interest in estimating the effect of important groupings or covariates on features of the circadian rhythm. These features can be characterized by an overall mean, amplitude (defined here as the distance from the lowest to the highest point in the rhythm), and phase shift (i.e., shifting of the whole pattern).

Others have proposed approaches for statistical analysis in ciradian rhythm longitudinal data [19,20]. In this paper, we develop an approach that allows investigators to compare circadian patterns on longitudinal count data in terms of amplitude, phase-shift, and overall mean, while adjusting for confounding factors.

Wang, Kee and Brown [21] and Albert and Hunsberger [22] proposed a regression-based approach for analyzing longitudinal continuous circadian rhythm data where individual variations were incorporated through random effects added to the mean, amplitude, and phase shift. The adaptation of this approach to the analysis of longitudinal circadian rhythm count data (as compared to continuous data) requires the development of new statistical methodology, which we develop in this paper.

## Materials and methods

We adapted Albert and Hunsberger’s approach to the Poisson regression framework. The goal was to develop a simple approach to estimating circadian rhythms that can be easily implemented by practitioners and can be used for analyzing longitudinal activity data.

The analysis is based on data obtained from the NEXT Generation Health Study. Data was collected by the *Eunice Kennedy Shriver* National Institute of Child Health and Human Development (NICHD) of the Nation Institutes of Health (NIH) in the summer of 2010. Analyses were performed using SAS version 9.2 software [23] and STATA version 11 [24]. Model fitting was done using PROC NLMIXED in SAS.

### Data

A nationally-representative cohort of U.S students in grade 10 was recruited using a multistage stratified design. Primary sampling units consisted of school districts or groups of school districts stratified across the nine U.S. Census divisions. Within this sampling framework 137 schools were selected and formally recruited; 80 (58.4%) agreed to participate. Tenth-grade classes were randomly selected within each recruited school and 3,796 students were recruited to participate; youth assent and parental consent were obtained from 2,619 (69.0%) students. Of those who consented, 2,519 (96.24%) completed the Wave 1 survey which was administered by trained research assistants. In a US nationally representative sample, the subsample of African American youth might not be sufficient to support robust statistical comparisons across race/ethnicity; therefore, additional African-American students were recruited into the sample. The prevalence of Hispanic youth in a national sample of this age group did not make it necessary to oversample Hispanic youth.

The study protocol was reviewed and approved by the Institutional Review Board of the *Eunice Kennedy Shriver* National Institute of Child Health and Human Development.

### Measures

Demographic information was provided by the adolescent and her parents. Race/ethnicity of the adolescent was classified into four categories: Hispanic, African-American, Asian, and White. In the analysis there were only three categories: Hispanic, African-American, and Other. Since there were only 2 Asians in the first classification, we combined them with the “White” category to create “Other”. The adolescent provided an estimate of family socioeconomic status using the Family Affluence Scale (FAS), a validated measure of socioeconomic status [25]. The adolescent answered four questions regarding material conditions of their household: “*Does your family own a car, van or truck*” (none = 0, 1 = 1, 2 or more = 2); “ *Do you have your own bedroom for yourself*” (no = 0, yes = 1); “ *During the past 12 months, how many times did you travel away on holiday with your family*”(not at all = 0, once = 1, twice = 2, more than twice = 3); and “ *How many computers does your family own*” (none = 0, one = 1, two = 2, more than two = 3). The FAS was used as a continuous scale in analyses with possible scores ranging from 0 to 9.

Adolescents’ primary caretakers reported their education level using a seven point scale: less than a high school diploma; high school diploma; GED; some college or technical school; associate’s degree; bachelor’s degree; or graduate degree. And, if applicable, reported education level of another parent or guardian providing support for the adolescent whether living with the adolescent or living separately.

Height was assessed using a portable stadiometer places on a level, hard surface. Students removed shoes and measures were taken at least twice. If the first two measures were not within ± 1.0 cm of each other, measurement was repeated. Failure to obtain two measures ± 1.0 cm of each other resulted in measures being repeated by a supervisor. Weight was measured using a Healthometer Model 498 KL Digital Scale on a hard, level surface. Students removed heavy objects from their pockets and extra outerwear (e.g., sweater, sweat shirt, or jacket). If the first two measurements were not within ± 0.2 kg, a third measurement was taken. Mean height and weight values of the two measures meeting the criteria were used to calculate Body Mass Index (BMI = wt(kg)/ht(m)^2^). Weight status (underweight, normal weight, overweight, and obese) were determined from BMI-for-age percentiles for each gender using the CDC 2000 growth chart [26]). Underweight was defined as a BMI below the 5^th^ percentile; normal weight was ≥ 5^th^ but < 85^th^ percentile; overweight was when BMI was ≥ 85^th^ but < 95^th^ percentile; and obese was ≥ 95^th^ percentile. For these analyses, overweight and obese were combined and are labeled ‘obese’ in subsequent descriptions and normal weight (‘non-obese’) was the comparison group.

Based on weight status, 281 overweight/obese and 286 normal weight adolescents were recruited from 40 of the participating schools for additional assessment procedures. Underweight adolescents were excluded. For one of the additional assessments, adolescents in both groups were asked to wear an Actiwatch2 on their non-dominant wrist for 24 hours/day for seven consecutive days. This device has been validated as a measure of activity in youth. The Actiwatch2 recorded motion in 30-sec epochs. Although the primary purpose of the Actiwatch2 is to assess sleep patterns, because it can easily be worn throughout the day, it provides an acceptable measure of activity during the day 23 [27].

A subsample of 96 female adolescents was selected for analysis in this paper (19 obese and 77 non-obese participants). The criteria for selection included: 1) having worn the Actigraph2 for a full 7 days; 2) the entire period of assessment was during the summer when school is not in session in order to avoid the effect of differences in school schedules; and 3) female (to avoid gender differences in activity level). Activity counts collected in the 30-sec epochs were summed over 15 min periods to provide 96 observations/day quantifying activity. This resulted in 672 observations within each student for 7 days.

### Models

We propose a shape-invariant Poisson model for circadian rhythm count data, which is an adaptation of Albert and Hunsberger [22] to the longitudinal activity example. Albert and Hunsberger developed a random effects shape invariant model that incorporates differences between individual circadian rhythms of cortisol, a highly predictable biological rhythm, by allowing important features, mean, amplitude and shift parameters, to vary based on fixed effect covariates and individual random effects. A direct extension to the Poisson outcome (or more generally to a generalized linear model outcome) would require the development of new non-standard software, and would be difficult to implement for the practitioner. We propose a simple two-stage approach that is simple for the practitioner to implement.

Let $y_{\mathrm{ij}}$ be activity counts for the $i^{\mathrm{th}}$student at the $j^{\mathrm{th}}$time; $\lambda_{\mathrm{ij}}$ is the mean activity for the $i^{\mathrm{th}}$student at the $j^{\mathrm{th}}$time for *i = 1,2,…I = 96; j = 1,2,…,J = 96*. We consider a model with $y_{\mathrm{ij}}$ as Poisson with mean$\lambda_{\mathrm{ij}}$ expressed as $\log\left(\lambda_{\mathrm{ij}}\right)=A_{i}+e^{-B_{i}}f\left(t_{\mathrm{ij}}-\varphi_{i}^{*}\right)$ in which $A_{i}$ is the overall average of activity of the $i^{\mathrm{th}}$ student, $e^{B_{i}}$ is relative change in amplitude for the $i^{\mathrm{th}}$ student around $A_{i}$, and $\varphi_{i}^{*}$ is the phase shift of activity of the $i^{\mathrm{th}}$ student. Note that a phase shift corresponds to a shift in the whole curve. The function $f\left(t-\phi_{i}^{*}\right)$ characterizes the common circadian pattern across students. This can be expressed as a harmonic function, parameterized as

(1) $$f\left(t\right)=\sum_{k=1}^{K}\beta_{k}\cos\left(2k\pi \left(t+\varphi_{i}^{*}\right)\right)$$

where $\beta_{1}=1$, *K* is the number of harmonics, and an increasing *K* results in an increasingly flexible circadian pattern. Note that $\beta_{1}=1$ is assumed in order for the model to be identifiable. For 2 harmonic terms, *f*( *t*) can be expressed as

(2) $$f\left(t\right)=\cos\left(2\pi \left(t+\varphi_{i}^{*}\right)\right)+D\cos\left(4\pi \left(t+\varphi_{i}^{*}\right)\right)\text{.}$$

In our analysis, we chose *K* = 2 (two harmonic terms) since this provided a flexible pattern that nicely characterized our data (i.e. mean curves from fitted models are close to the empirical means). Analyses with K = 3 provided similar results (data not shown). We used an inverse logit transformation for $\phi_{i}$, $\left(\text{i.e}.,\varphi_{i}^{*}=\frac{\exp\left(\varphi_{i}\right)}{1+\exp\left(\varphi_{i}\right)}\right)$ which restricts the phase shift parameter to be within the interval [0, 1], corresponding to characterizing the shift in terms of the percentage of a 24 hour period. For simplicity, the time variable of a 24-hour period (*t*) is rescaled to [0, 1]. For a typical day, one segment corresponds to 15 minutes from 12:00 AM to 12:15 AM (i.e., *t* = 1/96). The second segment corresponds to the time from 12:15 AM to 12:30 AM (i.e., *t* = 2/96), etc. There are 4 such segments in 1 hour and 96 segments in 24 hours. The distribution of mean, amplitude, and phase-shift parameters can be compared across populations using simple statistical methods such as two-sample tests and regression techniques.

Although the modeling framework assumes Poisson counts given individual parameters of intercept, amplitude, and phase-shift, the proposed estimation procedures can easily be altered by using a negative binomial model. However, the effect of covariates on changes on harmonic parameters using Poisson regression should be robust to the assumption of no overdispersion.

### Estimation

We propose a two-stage approach for estimation of the shape invariant model for longitudinal activity count data. In the first stage, subject-specific model parameters are estimated by iterating between fitting individual Poisson regression models and using a nonlinear optimizer which involve the 2 steps described below. In the second stage, we regress the individual model parameters on important covariates.

The first stage can be implemented as follows

Step 1: Estimate $A_{i}$, $B_{i}$, and $\phi_{i}$ foreach student forfixed D. This involves obtaining the maximum likelihood estimation through Poisson regresion on an individual by individual basis.

Step 2: Estimate D in (2) with estimated values of $A_{i}$, $B_{i}$, and $\phi_{i}$ from step 1 using maximum likelihood using nonlinear optimization.

We iterate between Step 1 and 2 until we have convergence.

In the second stage, we regress individual estimates obtained from stage 1 on covariates such as BMI. For the dichotomous single BMI covariate this simplifies to conducting a *t*-test on the three components characterizing the circadian pattern. A regression model can be applied when adjusting for confounding factors. Estimation of $A_{i}$, $B_{i}$, $\phi_{i}$ and D using the proposed two-step procedure may induce dependence in estimates across individuals, a violation of a key assumption in standard regression or the *t*-test. We investigate if any induced dependence between $A_{i}$, $B_{i}$, $\phi_{i}$ and D might affect the type I error rate of the statistical test.

A simulation was conducted to examine whether the two-side *t*-test in stage 2 results in valid hypothesis tests (i.e., an alpha-level procedure). More specifically, we were interested if the *t*-test was rejecting the null hypothesis of no significant BMI differences 5% of the time when BMI has no effect on the circadian pattern. We simulated 2000 circadian rhythm data sets. The simulation verified that the nominal α=0.05 is contained within a 95% confidence interval for the rejection rate for each parameter. That is, $\alpha_{A}$: (0.0395, 0.058), $\alpha_{B}$: (0.046, 0.066), $\alpha_{\varphi }$: (0.045, 0.065). Since, in each case, the interval contains 0.05, the two-stage approach appears to be a valid test.

## Results

A total of 96 subjects were included in the analysis. Table 1 shows important demographic patient information for these individuals.

**Table 1:** Demographic information

**Covariates**	**N (%) or Mean (SD)**
Race *	
1: Hispanic	35 (36.84%)
2: African American	26 (27.37)
3: Asian	2 (2.11%)
6: White	32 (33.68%)
	1 missing
Race (redefined) *	
1: Hispanic	35 (36.84%)
2: African American	26 (27.37)
3: Other	34 (35.79%)
	1 missing
BMI Group *	
0: Low BMI Group	77 (80.21%)
1: High BMI Group	19 (19.79%)
BMI **	25.45 (5.45); N= 96
Family Affluence **†	5.05 (1.61); N= 96
Parental Education 1 **††	3.70 (1.79); N= 92
Parental Education 2 **†††	3.36 (1.73); N= 61

* N (%) is the number and (percentage) for each level of categorical covariate.

** Mean (SD) is the mean and (standard deviation) of each continuous covariate.

Note: Race is redefined by combining the “Asian” group with “White” since there were only 2 Asians. This is what we used for the analysis.

† Family Affluence: validated socio-economic status.

†† Parental Education 1: the highest level of education completed by first guardian in the household.

††† Parental Education 2: the highest level of education completed by second guardian in the household if available.

All individuals contributed 96 segments of 15 minute activity count data for 7 days of the week. The two stage analysis was performed to assess the association between obesity (obese and non-obese) and the circadian rhythm of activity levels.

**Figure 1:**
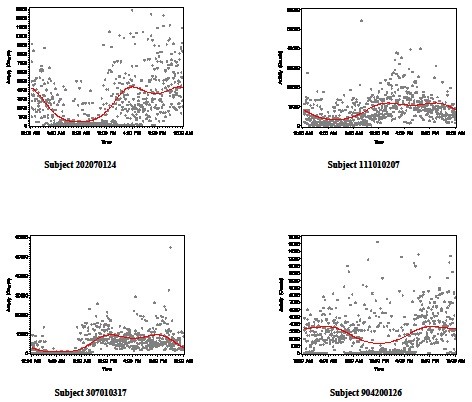
Average activity of 4 subjects.

At the first stage, we fit the activity data for each student and estimated individual parameters from the nonlinear shape invariant model (see the estimation procedure). At the second stage, we used the final estimates of $A_{i}$, $B_{i}$, and $\phi_{i}$ to assess the association between BMI groups and three components of the circadian pattern of activity. This model will permit us to examine whether BMI affects the students’ 24 hour activity pattern during the summer. Figure 1 displays individual plots of four students chosen at random showing the activity averaged at each 15 minute interval over 7 days (weekdays and weekends). For all individuals, we have 96 observations (four 15 minute intervals x 24 hours) per day. So, an average is taken over the 7 days. The plots demonstrate that there was sizable variation across individual students.

**Figure 2:**
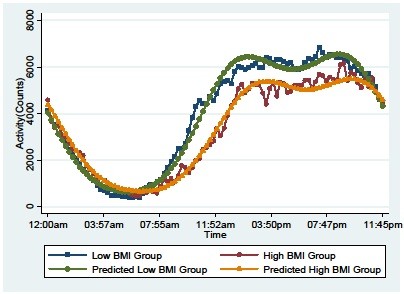
**Activity plots for non-obese (BMI: 17.78kg / m**^**2**^**– 28.60kg / m**^**2**^**) and obese participants (BMI: 29.16kg / m**^**2**^**– 47.98kg / m**^**2**^**).** Predicted means are estimated by the point-wise average of individual predicted means for each 15 minute interval.

Figure 2 shows the circadian pattern in activity over a 24-hour period by high and low BMI status. Differences in the circadian rhythm by BMI group can be characterized by differences in the overall mean, amplitude, and phase shift between groups.

A visual inspection of Figure 2 suggests that there was a shift in activity among the obese students. Specifically, obese adolescents tend to go to sleep and wake up later than non-obese adolescents. There also appears to be an overall lower mean activity level in obese as compared to non-obese adolescents. The proposed shape invariant statistical model for longitudinal activity count data can be used to formally test these empirical observations.

To compare the mean, amplitude and phase shift between the obese and non-obese participants, we performed a *t*-test for comparing $A_{i}$, $B_{i}$, and $\phi_{i}$. Using our shape invariant model, we fit the circadian rhythm for the BMI groups. The shape invariant model fit is superimposed on the observed plots (Figure 2). Table 2 shows the differences in mean level, amplitude, and phase shift between groups. There was a significant difference in the overall mean (*P* = 0.018) and phase shift ( *P* = 0.014) between the two BMI groups. The overall activity level (mean) was higher for non-obese as compared with obese girls. More interesting, obese girls had a circadian activity pattern that is significantly shifted later as compared with their non-obese counterparts.

**Table 2:** Differences in average circadian parameters between non-obese and obese participants

**Parameter**	**Difference Estimate (SE)**	***P***
*A*	0.21 (0.087)	0.018
*B*	-0.02 (0.074)	0.79
*ϕ*	1.13 (0.45)	0.014*

The parameters *A, B*, and *ϕ* denote mean values of individual parameters for either the non-obese or obese groups.

* One outlying observation from an obese participant with a value of -18.5 was deleted when estimating the difference and performing the *t*-test. A Wilcoxon rank sum test with this observation included was highly statistically significant ( *P* = 0.007).

We compared the difference in circadian activity patterns for obese and non-obese participants after adjusting for race, family affluence, and parental education. Differences in the phase-shift between groups remained statistically significant (*P* = 0.016), while differences in the mean and amplitude were not ( *P* = 0.19 and *P* = 0.75, respectively). In addition to the previous analysis where we dichotomizing BMI into two categories of obesity status, we performed an analysis treating BMI as continuous(17.78 *kg/m*^*2*^ to 47.98 *kg/m*^*2*^). The second stage of this analysis used a regression model as compared with a *t*-test. After adjusting for race, family affluence, and parental education, the phase shift increased with BMI ( *P* = 0.0072) but BMI was not related to either the mean amplitude ( *P* = 0.39 and *P* = 0.62, respectively).

## Discussion

We developed a new statistical approach to examine the effect of various factors on the circadian patterns in longitudinal count data. The modeling approach was developed with the aim of determining whether circadian patterns in activity are different between obese and non-obese teenagers. Our approach allowed us to focus the comparison on the mean, amplitude, and phase-shift of the circadian patterns. We found that, after adjusting for potentially confounding factors such as parental education and income as well as race, there was a statistically significant phase delay in the circadian timing of sleep and activity for obese versus normal weight adolescent girls. These results raise important scientific questions regarding the contribution of circadian phase abnormalities, changes in sleep time and timing and patterns of activity on overweight and obesity.

Although we found a statistically significant relationship between the phase-shift in activity and obesity, we cannot determine whether overweight girls develop a tendency to begin the day later or the shift in circadian patterns results in subsequent gains in weight. Change in sleep patterns (shifting later in day) have been associated with sleep deprivation [28] and sleep patterns have been related to weight gain in adolescents [29] and TV watching [30]. Much less is known about potential effects of shifts circadian patterns for physical activity.

Future work should examine whether these shifts in circadian patterns have implications for other health outcomes. For example, previous work has related adolescent morningness, or a tendency to prefer morning activities, with positive mental, social and physical health and adolescent eveningness with problem behaviors [31,32]. In the current study, the shift in the circadian pattern is also associated with less physical activity and relationships have been found between levels of adolescent physical activity and indicators of social, behavioral and physical health [33].

The new statistical methodology allowed us to focus attention on important features of the circadian pattern and find this interesting association. The larger overall activity and phase shift among obese as compared with non-obese adolescents raises important questions about the association between circadian phase, activity and sleep patterns and obesity. An overall increase in activity in normal weight adolescent girls could be attributed to a healthier pattern of activity and increased capacity to engage in a range of activities, thereby contributing to normal weight. There are several explanations for the phase shift between obese and non-obese adolescents. A mis-match between circadian phase and the timing of sleep may be present in this sample of obese adolescents that could result in abnormalities in the hormones that regulate appetite, energy metabolism and adipose deposition. It is also possible that decreased energy expenditure during the day and night and the phase shift could be an indicator of abnormalities or differences in the metabolic rate. It is not clear whether this phase shift is a precursor or a manifestation of obesity or a shared marker of a metabolic type or abnormality. Also, it is possible that the delayed activity pattern among obese girls may be associated with factors that were not controlled for in this analysis. For example, in the NEXT study we did not collect information about whether participants had summer jobs or scheduled activities or whether they could sleep on an ad hoc basis. As this appears to confirm that this approach has some promise, it will be important to evaluate other contributors to circadian phase and lifestyle differences in subsequent studies.

## Competing interests

The authors declare that they have no competing interests.

## Authors’ contributions

SO: Developed methodology, conducted analysis, drafted manuscript. PSA: Developed methodology, conducted analysis, drafted manuscript. DL: Design study, drafted manuscript. RJI: Design study, drafted manuscript. All authors read and approved the final manuscript.

## Acknowldegments

This work was supported by the Intramural Research Program of the *Eunice Kennedy Shriver* National Institute of Child Health and Human Development and the National Heart Lung and Blood Institute.
